# Generation of a new thermo-sensitive genic male sterile rice line by targeted mutagenesis of *TMS5* gene through CRISPR/Cas9 system

**DOI:** 10.1186/s12870-019-1715-0

**Published:** 2019-03-20

**Authors:** Hirendra Nath Barman, Zhonghua Sheng, Sajid Fiaz, Min Zhong, Yawen Wu, Yicong Cai, Wei Wang, Guiai Jiao, Shaoqing Tang, Xiangjin Wei, Peisong Hu

**Affiliations:** 1 State Key Laboratory of Rice Biology, China National Rice Research Institute, Hangzhou, 310006 China; 2 Plant Physiology Division, Bangladesh Rice Research Institute, Gazipur, Bangladesh

**Keywords:** Gene editing, Heterosis, Thermo-sensitive genic male sterility (TGMS), *TMS5* gene, Two-line hybrid rice system

## Abstract

**Background:**

Two-line hybrid rice with high yield potential is increasingly popular and the photo- and temperature-sensitive male sterile line is one of the basic components for two-line hybrid rice breeding. The development of male sterile lines through conventional breeding is a lengthy and laborious process, whereas developing thermo-sensitive genic male sterile (TGMS) lines for two-line hybrid breeding by editing a temperature-sensitivity gene by CRISPR/Cas9 is efficient and convenient.

**Results:**

Here, thermo-sensitive genic male sterility (TGMS) was induced by employing the CRISPR/Cas9 gene editing technology to modify the gene *TMS5.* Two TGMS mutants, *tms5–1* and *tms5–2*, both lacking any residual T-DNA, were generated in the *indica* rice cultivar Zhongjiazao17 (cv. YK17) background. When grown at a sub-optimal temperature (22 °C), both mutants produced viable pollen and successfully produced grain through self-fertilization, but at temperatures 24 and 26 °C, their pollen was sterile and no grain was set. F_1_ hybrids derived from the crosses between YK17S (*tms5–1*) and three different restorer lines outperformed both parental lines with respect to grain yield and related traits.

**Conclusion:**

The YK17S generated by CRISPR/Cas9 system was proved to be a new TGMS line with superior yield potential and can be widely utilized in two-line hybrid breeding of *indica* rice.

**Electronic supplementary material:**

The online version of this article (10.1186/s12870-019-1715-0) contains supplementary material, which is available to authorized users.

## Background

Rice (*Oryza sativa* L.) is a staple crop for a major fraction of the world’s population, so increasing its productivity is a priority for ensuring global food security. The heterosis expressed by F_1_ hybrid rice cultivars translates into a grain yield advantage of 10–20% over conventional inbred cultivars, therefore F_1_ hybrid rice has come to occupy about 60% of the rice production area in China [1], and being cultivated in over 40 countries [2]. F_1_ hybrid grain can be produced using either a three-line or a two-line system. The former requires three genetic stocks, namely a male sterile line (typically expressing cytoplasmic genic male sterility), a maintainer line and a restorer line [3], while the latter exploits male sterility which is only induced by a specific environmental condition (either temperature or photoperiod or both) [4]. The two-line system is more cost-effective, more flexible in terms of germplasm, produces higher quality grain and is simpler to operate than the three-line system [5]. A number of genes have been identified as suitable for two-line hybrid rice breeding: these include the thermo-sensitive genic male sterility (TGMS) genes *TMS1*-*TMS10* and the photoperiod-sensitive genic male sterility (PGMS) genes *PMS1*-*PMS3* [6, 7]. The first PGMS line to be bred (in 1973) used the *japonica* type cultivar Nongken58, and was based on a loss-of-function mutant of *PMS3* [8]. Another *pms3* line, bred in the *indica* type cultivar Pei’ai 64S, has been widely used by breeders [9]. In China, loss-of-function mutants of *TMS5* have made major contribution to two-line hybrid rice breeding; about 71% of cultivars generated by this method possess the *tms5* background [10, 11], starting with the TGMS line AnnongS-1, bred in 1987 [12–14]. Another *tms5* line Zhu1S is also a widely used source of male sterility [11].

Male sterile lines developed through conventional breeding as well as by marker assisted selection (MAS) are a lengthy and laborious processes. With the advent of modern tools such as CRISPR/Cas9, the breeding time can be shortened significantly [15]. The CRISPR/Cas9 gene editing system developed in 2013 has proved to be an effective technology for generating targeted mutations in a wide array of cells and organisms [16]. This system has been shown to be effective in major cereals and successfully deployed to develop powdery mildew resistant wheat [17], glutinous maize [18] and TGMS maize [19] by targeted mutagenesis of the *MILDEW-RESISTANCE LOCUS (MLO)*, *Waxy* and *ZmTMS5* genes, respectively. The glutinous [20], high-amylose [21], fragrant [22], sweet endosperm [23], blast resistant [24], herbicide resistant [25] and nitrogen using efficiently [26] rice germplasms have also been developed by editing the *Waxy*, *SBEIIb*, *Badh2*, *ISA1*, *OsERF922*, *ALS* and *NRT1.1B* genes, respectively.

In the present study, we reported targeted mutagenesis of *TMS5* gene in an elite *indica* cultivar Zhongjiazao17 (cv. YK17). YK17 is a popular and conventional cultivar vastly cultivated in southern region of China. This cultivar corresponds to an early maturing, moderate plant type, has a higher seed-setting rate and yield, and is resistant to rice blast and white leaf blight. A novel TGMS line, YK17S, which possesses critical sterility inducing temperature (CIST) < 24 °C was developed. YK17S was crossed with three restorer lines and all F_1_ hybrids outperformed both parental lines in terms of grain yield and related traits.

## Results

### Mutation of *TMS5* via CRISPR/Cas9

**Fig. 1:**
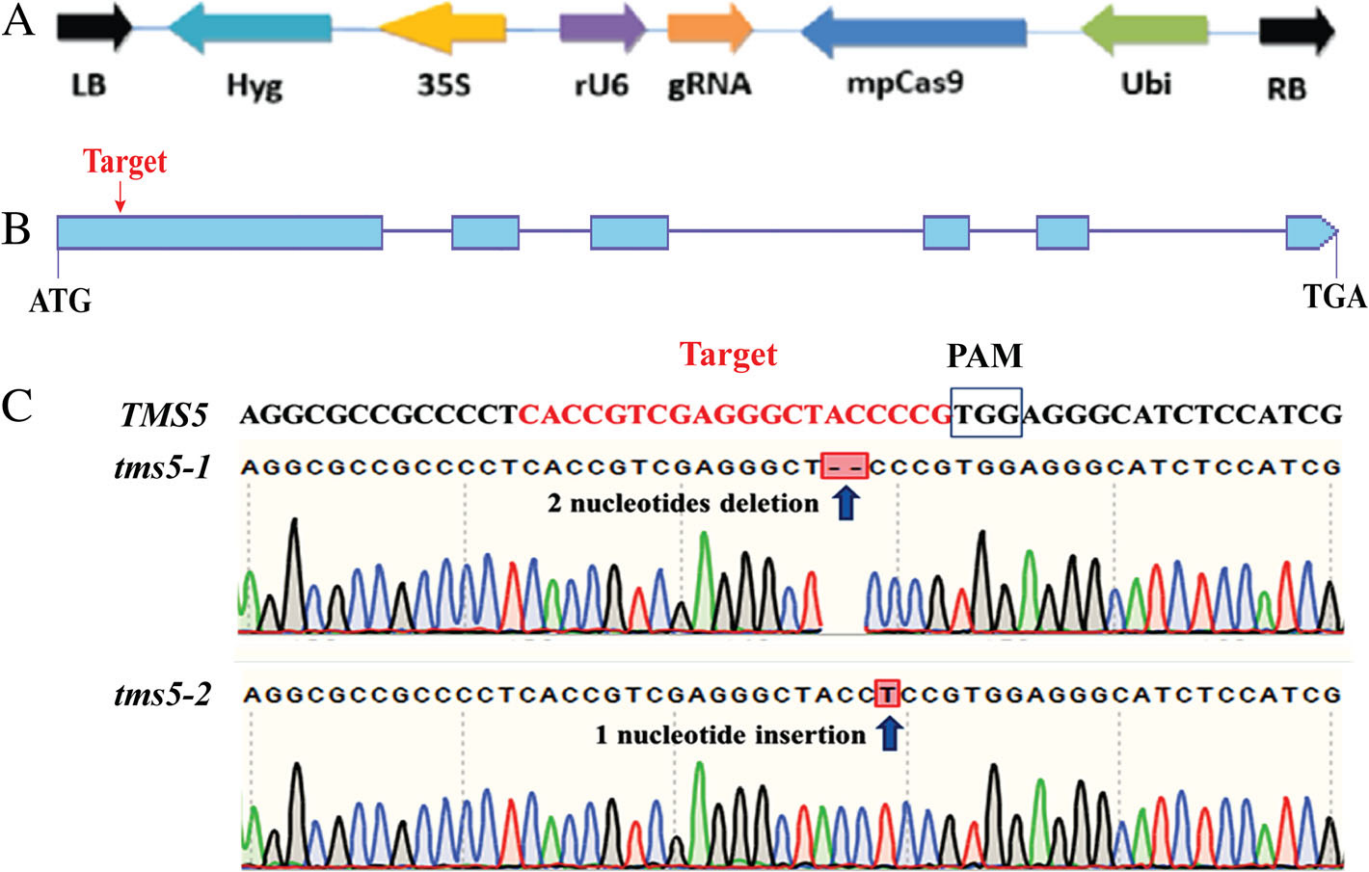
CRISPR/Cas9-mediated editing of *TMS5*. **a** Vector structure of Cas9/gRNA (VK005–01). **b** Structure of *TMS5*. **c** The *TMS5* target site aligned with the *tms5–1* (a 2 nucleotides deletion) and the *tms5–2* (1 nucleotide insertion) mutant sequences. LB: vector left border, Hyg: Hygromycin, 35S: CaMV 35S promoter, rU6: rice U6 promoter, gRNA: guide RNA, mpCas9: Cas9 protein, Ubi: *Ubiqutin* promoter, RB: vector right border, PAM: protospacer adjacent motif

**Fig. 2:**
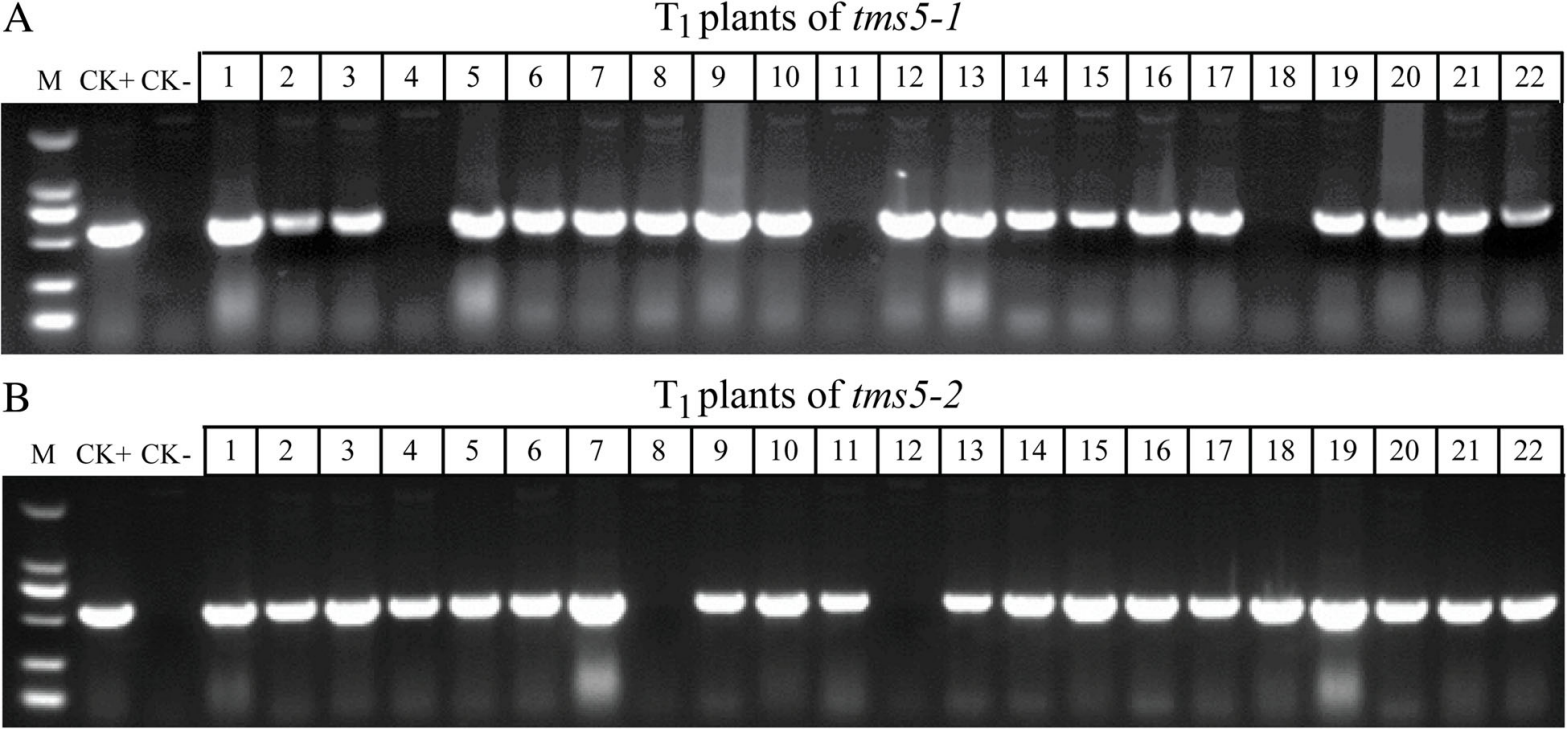
PCR-based identification of T-DNA-free T_1_ segregants using primers directed at the *hpt* sequence. Segregants among (**a**) *tms5–1* plants, and (**b**) *tms5–2* plants. VK005–01 was used as the positive control (CK+) and cv. YK17 as the negative control (CK-). M: 2 kbp DNA ladder

The CRISPR/Cas9 vector used in the study contained a gRNA sequence, that included a target site within the first exon of *TMS5*, and was driven by the rice U6 promoter **(**Fig. 1a and b). The construct was inserted into cv. YK17 via *Agrobacterium*-mediated transformation. Two types of homozygous mutations were detected in the T_0_ generation: *tms5–1* was the result of a 2 nucleotides deletion and *tms5–2* with a 1 nucleotide insertion **(**Fig. 1c). The segregation of T-DNA was noted in both of the two T_1_ families, as confirmed by PCR **(**Fig. 2), but the genomic sequence of three *tms5–1* mutants (plants #4, #11 and #18) and of two *tms5–2* segregants (plants #8 and #12) were free of any T-DNA. A scan for off-target mutations in all five T-DNA-free plants showed that none of the likely off-target sites in different chromosomes were affected (Additional file 1: Table S1). Therefore, the five T-DNA-free plants were advanced to multiply the T_2_ generation for further research.

### Morphological features of the *tms5* mutants

**Fig. 3:**
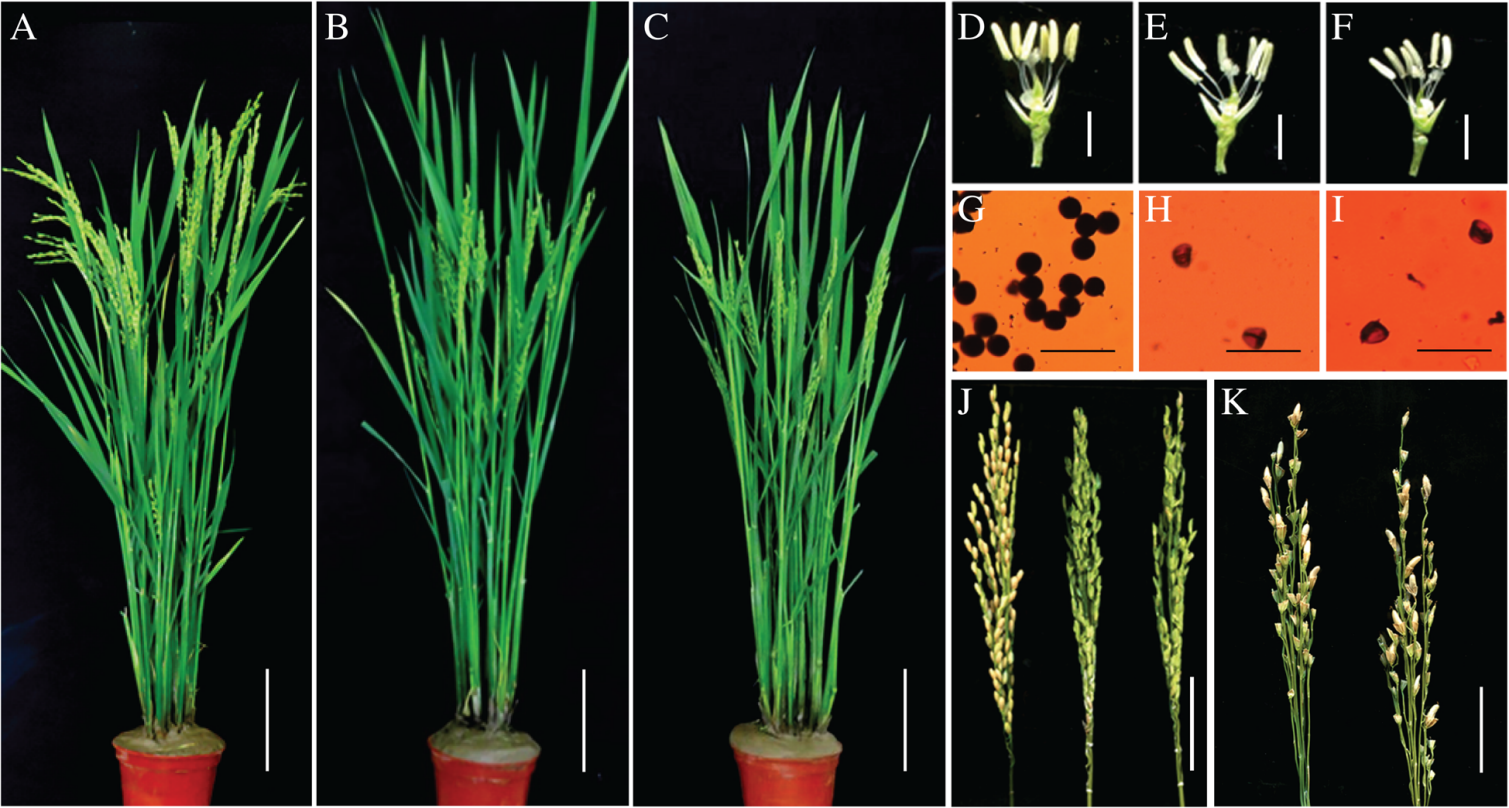
Phenotype of cv. YK17 and T_2_ generation of *tms5–1* and *tms5–2* grown in the field during the normal rice growing season. **a**-**c** Appearance of the whole plant. **d**-**f** Spikelets after removal of the lemma and palea. **g**-**i** Pollen grains stained with I_2_-KI. **j** Panicles of cv. YK17, *tms5–1* and *tms5–2* from left to right, respectively. **k** Grain set by *tms5–1* (left) and *tms5–2* (right) after manual pollination. Bar in A-C: 15 cm, D-F: 0.5 cm, G-I: 100 μm, J: 5 cm, K: 3 cm

With regards to the plants grown in the field during the normal growing season, the only discernible phenotypic difference between the *tms5* selections and cv. YK17 was about 5 cm reduction in plant height (Fig. 3a-c; Additional file 1: Figure S1). The floral organs of the mutants and cv. YK17 were indistinguishable **(**Fig. 3d-f), but the anthers of both mutants contained either no pollen or just a few sterile pollen grains, resulting in the plants’ self-sterility (Fig. 3g-j; Additional file 1: Figure S1E). The viability of the megagametophyte of the *tms5* mutants was confirmed by manual crossing using the normal pollens, and all crosses developed healthy grains (Fig. 3k).

### The effect of temperature on pollen production in the *tms5* mutants

**Fig. 4:**
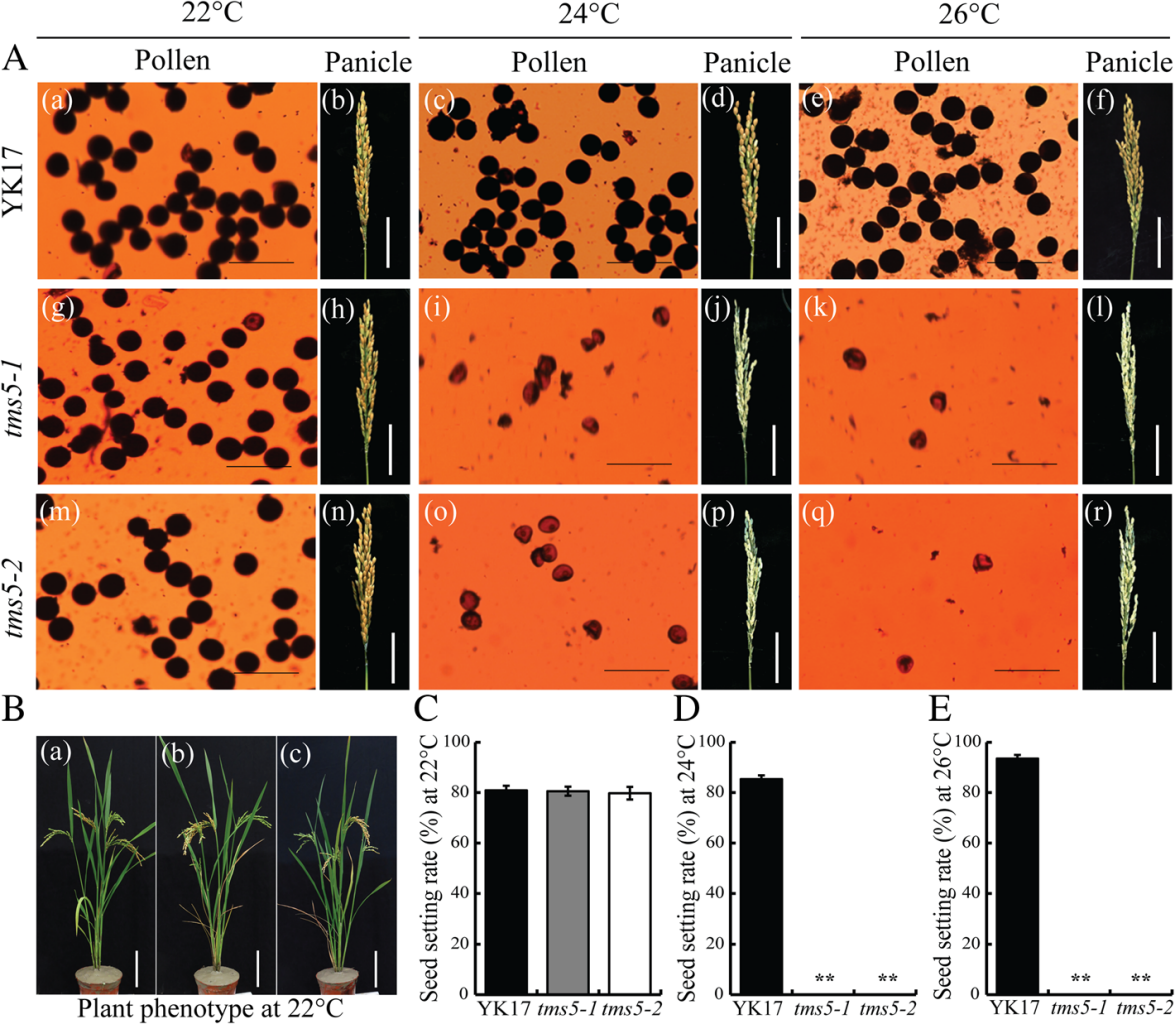
Pollen fertility and grain set of cv. YK17 and T_2_ generation *tms5–1* and *tms5–2* plants grown under various daily average regimes. **a** [a-f] cv. YK17, [g-l] *tms5–1,* [m-r] *tms5–2* plants exposed to 22 °C, 24 °C or 26 °C. **b** Appearance of the whole plant of (a) cv. YK17, (b) *tms5–1*, (c) *tms5–2* grown at 22 °C. **c**-**e** Grain set at 22 °C, 24 °C or 26 °C. Values are shown as mean ± SD (*n* = 20). Asterisks (* *P* < 0.05; ** *P* < 0.01) indicate statistically significant differences between means of a mutant and cv. YK17, as determined by a student’s *t*-test. Bars in (A) [a, c, e, g, i, k, m, o, q]: 100 μm; [b, d, f, h, j, l, n, p, r]: 5 cm; in B: 15 cm

**Fig. 5:**
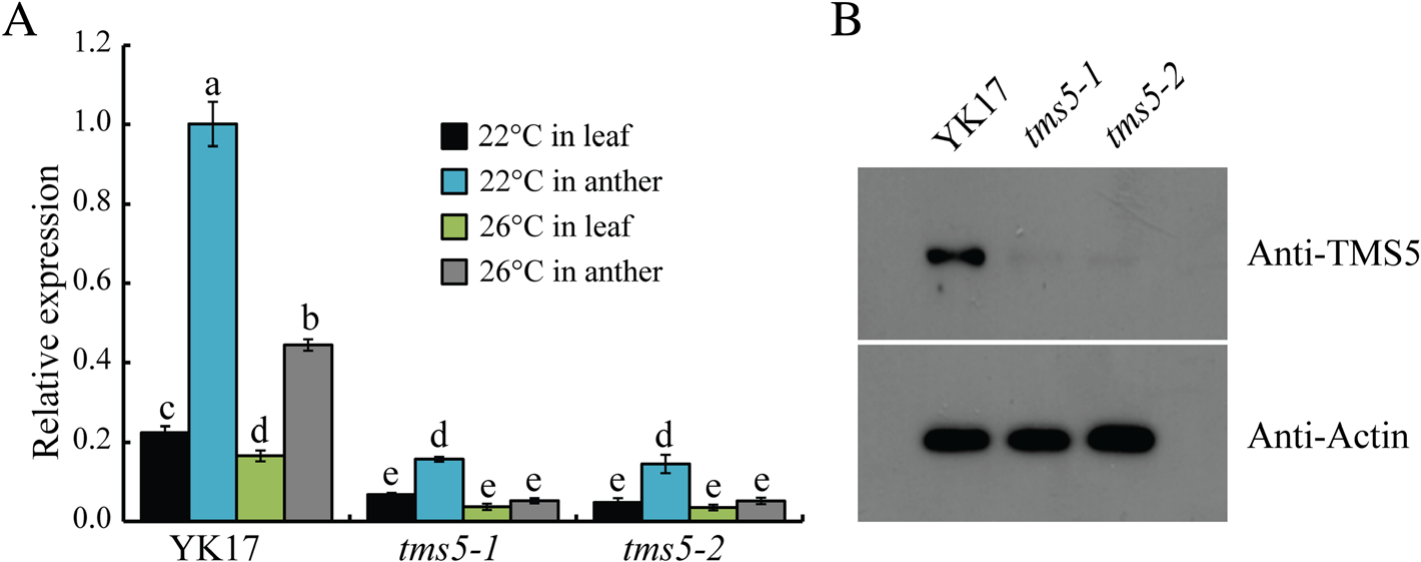
*TMS5* expression in cv. YK17 and T_2_ generation of *tms5–1* and *tms5–2* plants. **a** Transcript abundance as estimated by qRT-PCR in the leaves and anthers of plants grown at either 22 °C or 26 °C. Values shown in the form mean ± SD (*n* = 3). Columns marked with a different letter indicate statistically significant differences between means (*P* < 0.05). **b** Western blot assay of TMS5 in the panicle of plants grown at 26 °C

Determining the suitable critical temperature for expression of sterility or fertility is essential when exploiting a novel TGMS line in heterosis breeding. The effect of varying the temperature on pollen production of *tms5* mutants (T_2_ generation) and cv. YK17 was tested in a growth cabinet supplying a daily average temperature of either 22 °C, 24 °C or 26 °C. The fertility of cv. YK17 plants was complete at all three temperatures resulting in grain set (Fig. 4a); however, both *tms5–1* and *tms5–2* plants were male sterile when exposed to either 24 °C or 26 °C with no grain set, while they became fertile when exposed to 22 °C and setting grains (Fig. 4a-e). At this lower temperature (22 °C), grain set was 81.3% for cv. YK17, 80.4% for *tms5–1* and 79.8% for *tms5–2* (Fig. 4c). The phenomenon persisted over successive generations of both *tms5–1* and *tms5–2* (Additional file 1: Figure S2). Expression analysis of *TMS5* in cv. YK17 and mutants plants grown at either 22 °C or 26 °C showed that the abundance of *TMS5* transcript in both the leaves and the anthers was much higher at the lower temperature, and was considerably higher in YK17 than its abundance in either of the mutants (Fig. 5a). At the protein level, a western blot assay indicated that the abundance of TMS5 in the panicle of YK17 was similarly much higher grown at 26 °C, whereas protein signals were barely detected in both two mutants (Fig. 5b). Thus both mutants were considered to be viable candidates to be used in a TGMS two-line breeding system designed to produce hybrid rice. The two *tms5–1* and *tms5–2* lines were designated as YK17S1 and YK17S2, respectively.

### The application of YK17S1 in two-line hybrid breeding

**Fig. 6:**
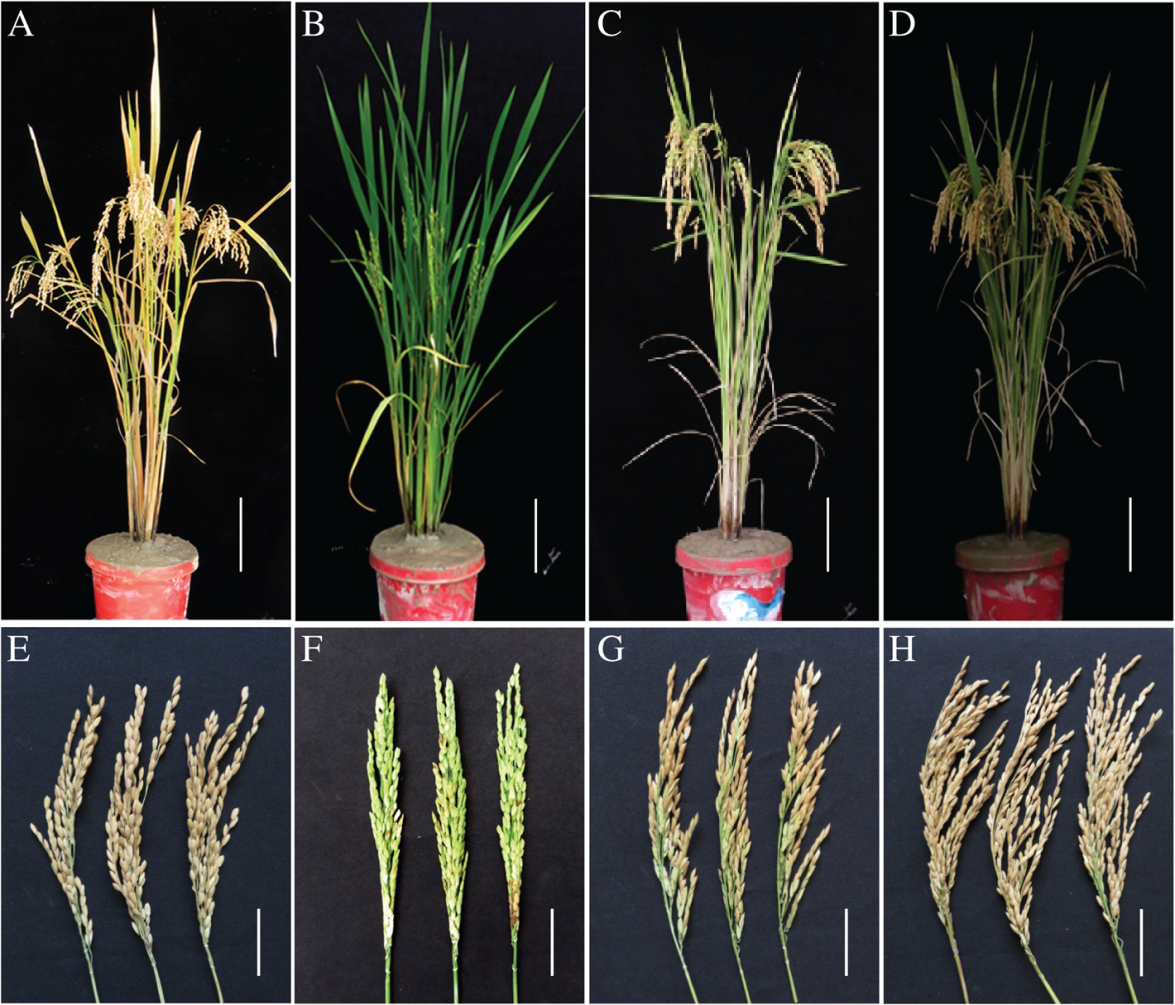
Phenotype of cv. YK17, YK17S1, R101 and the F_1_ hybrid of YK17S1 x R101. Appearance of the (**a**-**d**) whole plant and (**e**-**h**) panicle of cv. YK17, YK17S1, R101 and the F_1_ hybrid of YK17S1 X R101 from left to right, respectively. Bar in A-D: 15 cm, E-H: 5 cm

**Fig. 7:**
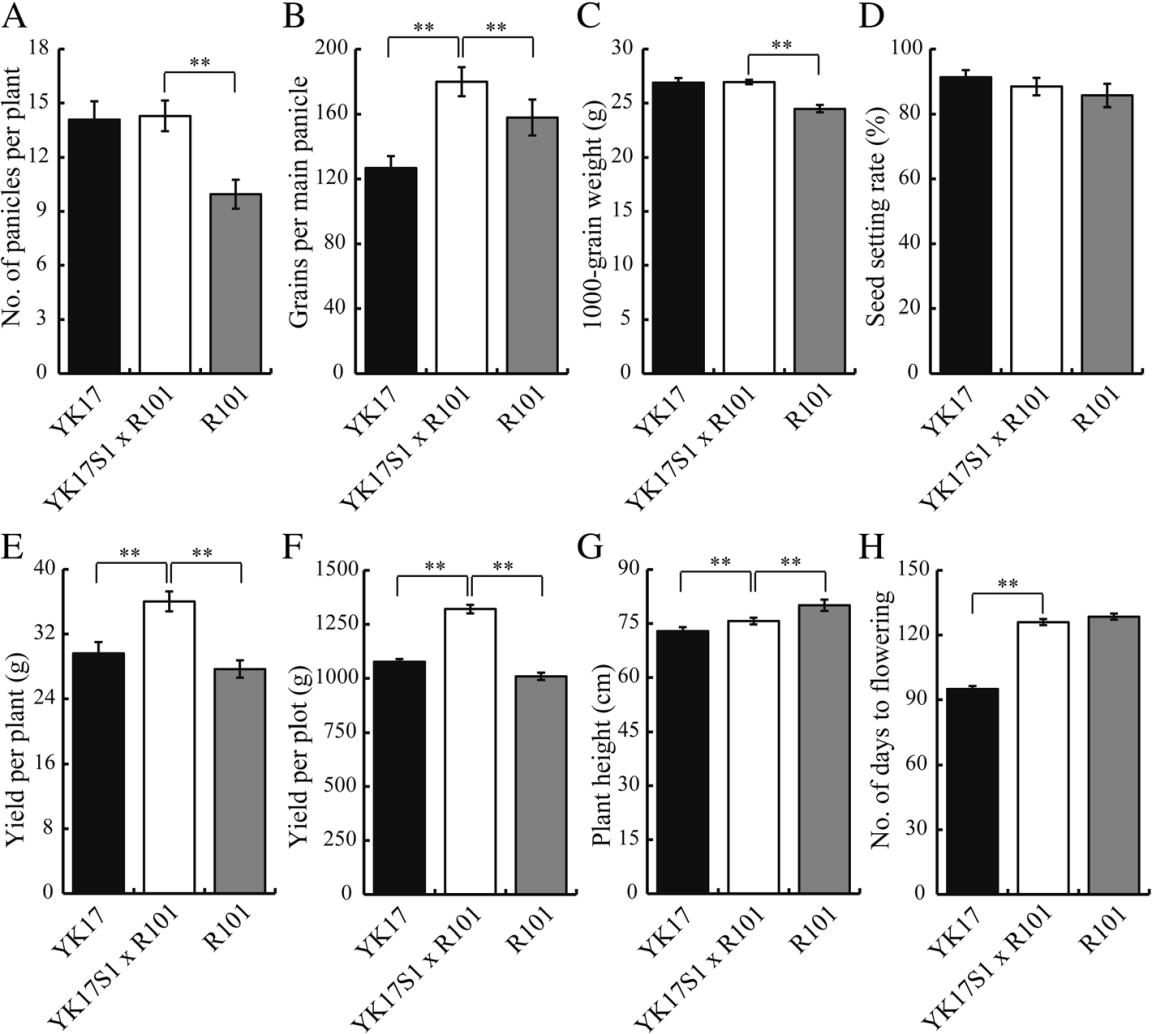
Grain yield performance and yield components of cv. YK17, YK17S1, R101 and the F_1_ hybrid YK17S1 x R101. **a** Number of panicles per plant, **b** number of grains set on the main panicle, **c** thousand grain weight (g), **d** grain set (%), **e** yield per plant (g), **f** yield per plot (g), **g** plant height (cm) and **h** number of days to flowering. Values shown in the form mean ± SD (**a**, **b**, **d**, **g**: *n* = 20; **c**: *n* = 4, **e**: *n* = 6, **f**, **h**: *n* = 2). Asterisks (* *P* < 0.05; ** *P* < 0.01) indicate statistically significant differences between the mean performances of either cv. YK17 and YK17S1 x R101, or R101 and YK17S1 x R101, as determined by a student’s *t*-test

The new TGMS line YK17S1 was crossed with the three different *indica* type restorer lines R101, R106 and R207, and the resulting hybrids (F_1_), along with the parental lines and cv. YK17, were grown in the field during the normal rice-growing season. All three hybrids (F_1_) out-performed both their parental lines and cv. YK17 in terms of major agronomic traits, including number of grains per panicle, grain yield per plant and grain yield per plot (Figs. 6 and 7; Additional file 1: Figures. S3-S6). The number of grains per panicle increased in the YK17S1 x R101 hybrid by 41.9% over that achieved by cv. YK17, by 35.8% in the YK17S1 x R106 hybrid and by 39.2% in the YK17S1 x R207 hybrid. The increases in grain yield per plant were 21.7, 16.3 and 17.4%, respectively, whereas that of grain yield per plot were 22.7, 17.1 and 18.4%, respectively. Although the number of panicles formed per plant and the thousand grain weight were also significantly higher in the hybrids than in either parent, there was no significant gain in either the rate of grain set, plant height or the number of days to heading (Fig. 7; Additional file 1: Figures. S5 and S6). In addition, although T-DNA was examined in F_1_ hybrid, following segregation, no T-DNA existed in all three hybrids detected (Additional file 1: Figures. S7-S9). In conclusion, the YK17S1 line represents a new valuable TGMS source and can be widely used in two-line hybrid breeding of *indica* rice.

## Discussion

The CRISPR/Cas9 system is proving to be an effective tool for site-specific genome editing [24]; the system is relatively simple to operate and induces a significant mutation rate [27–29]. A further advantageous feature is that the system gives the opportunity to select for plants which no longer harbor any T-DNA sequence [30], so that these materials – unlike genetically modified organisms (GMOs) - although created using transgenic technology, do not retain any foreign DNA. Crop varieties created using CRISPR/Cas9 technology can now be marketed in the USA without extensive regulatory monitoring [31]. Plant varieties with edited genome are starting to revolutionize the GMOs debate as the method for crop improvement is proving to be a safe approach compared to other genetic engineering technologies.

Male sterility performs a unique role in cytoplasmic male sterility (CMS) and in photoperiod /thermo-sensitive genic male sterility (P/TGMS) for hybrid breeding programs [3]. According to a recent report, several *tms5* mutants generated in a *japonica* type background using the CRISPR/Cas9 system exhibited a high level (85.3%) of pollen sterility when the plants were cultivated under a high temperature regime [15]. Ideally, the production of F_1_ hybrid seed/grain requires a level of sterility to be as close as possible to 100%. A low critical sterility inducing temperature (CSIT) is needed to ensure the purity of the hybrid seed produced in two-line hybrid breeding programs; and generally 23 °C is seen as suitable for temperate zones and 24 °C in subtropical zones [32, 33]. Both YK17S1 and YK17S2 were fully male fertile when were grown under a 22 °C regime, but were sterile at 24 °C, so their CSIT must lie between 22 °C and 24 °C. The precise temperature probably depends on the genetic background in which the mutation is present (in this case cv. YK17) [15]. When YK17S1 was crossed with three restorer lines, all three F_1_ out-performed their parents with respect to yield and its components, showing that YK17S1 has a wide range of compatibility. Altogether, our findings proved that YK17S1 is a novel *indica* background TGMS which can serve as an important source for speeding up heterosis breeding as well as commercial hybrid breeding in rice.

In addition, the present study demonstrated that targeted mutagenesis of *TMS5* gene using CRISPR/Cas9 in elite cultivars to develop TGMS line for two-line hybrid breeding was very efficient and convenient. In the future, TGMS lines having disease resistant, better yield and quality can also be developed by editing *TMS5* as well as other genes related to quality and disease at the same time in an appropriate genetic background. This strategy will boost up the development of excellent TGMS lines for two-line hybrid breeding.

## Conclusion

Targeted modification of *TMS5* by CRISPR/Cas9 system is a successful approach to develop TGMS lines for hybrid rice production. The developed TGMS line YK17S fulfilled the criteria of a typical male sterile line necessary for two-line hybrid system. The YK17S showed good compatibility in two-line hybrid rice breeding.

## Methods

### Plant materials and growing conditions

Plants of T_0_ and T_1_ generation were grown in growth cabinets maintaining at daily average temperature (DAT) ~ 22 °C to multiply seeds. T_2_ generation plants including wild-type cv. YK17 (Zhongjiazao17, an *Oryza sativa indica* cultivar bred by the China National Rice Research Institute) were cultivated in the field during the normal rice growing season at the China National Rice Research Institute (Fuyang, Hangzhou: 30°03′N, 119°57′E; with a DAT of 25–32 °C during the growing period). To test for the effect of temperature on pollen production, T_2_ and T_3_ plants, including cv. YK17, were grown in growth cabinets with a DAT of 22 °C, 24 °C or 26 °C and a 13.5 h photoperiod. The experiment comprised two replicates of each line. The F_1_ hybrid along with the parental lines and cv. YK17, were cultivated in the field during the normal rice growing season at the China National Rice Research Institute research station (Lingshui, Hainan: 18°30′N, 110°01′E).

### Vector construction and rice transformation

The target site (5′-CACCGTCGAGGGCTACCCCGTGG-3′) consist of a protospacer adjacent motif (PAM) lying within the *TMS5* coding sequence (*Os02g12290*). A BlastN search was conducted to ensure the uniqueness of the site. The gRNA target sequence was inserted into the VK005–01 vector (Viewsolid Biotech, China, http://www.v-solid.com) which harbors the rice U6 promoter. The resulting CRISPR/Cas9 construct was introduced into cv. YK17 by agroinfection [34]. After about two months of culture, transgenic regenerants were transferred to a growth cabinet. The sequences of the primers used in vector construction and identification are listed in Additional file 1: Table S2.

### Mutation detection and analysis of transgenic plants

Genomic DNA was extracted from the leaves of transformed plants using the sodium dodecyl sulfate (SDS) method [35]. PCRs amplifications were performed using primer pairs which generated an amplicon harboring the target site, and the resulting amplicons were sequenced using the Sanger method. Mutations were identified by comparing the amplicon sequences derived from putative transgenic and cv. YK17 templates. Homozygosity/heterozygosity for a mutated sequence was inferred from the chromatogram trace. T_1_ segregants homozygous for a *tms5* mutation were screened for the presence/absence of T-DNA using a PCR assay directed to the *hpt* sequence using the VK005–01 plasmids and cv. YK17 gDNA as positive and negative controls, respectively. The *hpt*-negative plants were considered T-DNA-free plants. The presence of any off-target mutations was monitored in plants lacking any T-DNA. Ten plants were randomly selected from the three F_1_ hybrids to test for the presence/absence of T-DNA by PCR assay. The relevant PCR primers for these steps are listed in Additional file 1: Table S2.

### Phenotypic analysis of T-DNA-free segregants

The rice florets of cv. YK17 and *tms5* mutant plants were collected during the anthesis period. Mature pollen grains were stained using 1% I_2_–KI solution and images were obtained using a DM1000 microscope (Leica, Germany). Rice florets were photographed using a Perfection V33 scanner (Epson, Japan).

### Transcription profiling and western blotting

Total RNA from leaves and anthers of plants grown under either a 22 °C or a 26 °C regime was isolated using the Trizol reagent (Invitrogen, USA, https://www.thermofisher.com). A 2 μg aliquot of RNA per sample was used to synthesize cDNA, using a ReverTra Ace qPCR RT kit (Toyobo, Japan, http://www.bio-toyobo.cn), according to the manufacturer’s protocol. The resulting cDNAs were used as templates for a quantitative real-time PCR (qRT-PCR) assay on the LightCycler 4.80 real-time PCR system (Roche, Switzerland), and using a SYBR Green Real-time PCR Master Mix (Toyobo). Relative transcript abundances were derived using the 2^-ΔΔCT^ method [36], the rice *Ubiquitin* gene (*Os03g0234200*) was used as the reference. The primer sequences used for the qRT-PCR are listed in Additional file 1: Table S2. For the western blot assay, total rice protein was extracted from about 0.1 g of panicles ground to powder in liquid nitrogen and homogenized with 1 ml extraction buffer [25 mM Tris-HCl (pH 7.4), 150 mM NaCl (pH 8), 1 mM EDTA, 1% Nonidet P-40, 5% glycerol and protease inhibitor (0.2% in working solution; Roche)]. The mixture was vortexed, and then chilled on ice for 30 min. Cell debris was removed by centrifugation (12,000 x *g* for 15 min at 4 °C). Protein separation, transfer to a membrane, hybridization and detection were performed using methods described in [37]. The membranes were probed with an anti-Actin antibody to ensure equal loading.

### Performance of F_1_ hybrids obtained from YK17S1

The TGMS line YK17S1 was crossed with three potential *indica* type restorer lines namely R101, R106 and R207 (bred by the China National Rice Research Institute). Panicles were covered using brown paper bags to avoid off-target pollination. F_1_ seeds were collected after 25–28 days when grains matured. The resulting F_1_ hybrids were cultivated in field plots, with the experiment set out as a replicated complete block design with 36 plants per plot, and each plot replicated twice. Plot borders were planted by purple rice to avoid border effects on agronomic traits.

## Additional file

Additional file 1: **Figure S1.** Agronomic performance of cv. YK17 and T_2_ generation *tms5–1* and *tms5–2* mutants grown during a normal growing season. **Figure S2.** Pollen fertility and grain set of cv. YK17 and the T_3_ generation mutants (YK17S1 and YK17S2) plants grown under various temperature regimes. **Figure S3.** Phenotype of cv. YK17, YK17S1, R106 and the F_1_ hybrid of YK17S1 x R106. **Figure S4.** Phenotype of cv. YK17, YK17S1, R207 and the F_1_ hybrid of YK17S1 x R207. **Figure S5.** Grain yield performance and yield components of cv. YK17, YK17S1, R106 and the F_1_ hybrid YK17S1 x R106. **Figure S6.** Grain yield performance and yield components of cv. YK17, YK17S1, R207 and the F_1_ hybrid YK17S1 x R207. **Figure S7.** PCR-based identification of T-DNA-free in F_1_ progenies of YK17S1 x R101 using primers directed at the *Cas9* (A), gRNA scaffold (B) and *hpt* (C) sequence. **Figure S8.** PCR-based identification of T-DNA-free in F_1_ progenies of YK17S1 x R106 using primers directed at the *Cas9* (A), gRNA scaffold (B) and *hpt* (C) sequence. **Figure S9.** PCR-based identification of T-DNA-free in F_1_ progenies of YK17S1 x R207 using primers directed at the *Cas9* (A), gRNA scaffold (B) and *hpt* (C) sequence. **Table S1.** Detection of mutations in potential off-target sites in T-DNA-free T_1_ generation segregants. Nucleotides corresponding to the protospacer adjacent motif in each target site are shown in red. Mismatches are shown in blue and matches in black. **Table S2.** List of primers used in the present study. (PDF 1285 kb)

## Abbreviations

CIST: Critical sterility inducing temperature
CMS: Cytoplasmic genic male sterility
CRISPR: Clustered regularly interspaced short palindromic repeats
DAT: Daily average temperature
GMOs: Genetically modified organisms
HPT: Hygromycin phosphotransferase
PAM: Protospacer adjacent motif
SDS: Sodium dodecyl sulfate
TGMS: Thermo-sensitive genic male sterility
TMS5: Thermo-sensitive genic male sterile 5
