# Supplementary material for: Generation of a new thermo-sensitive genic male sterile rice line by targeted mutagenesis of TMS5 gene through CRISPR/Cas9 system
Source: BMC Plant Biol. 2019 Mar 20;19:109. doi: 10.1186/s12870-019-1715-0 (PMC6425558; doi:10.1186/s12870-019-1715-0)
Supplement: Supplementary file 1 — Figure S1. Agronomic performance of cv. YK17 and T2 generation tms5–1 and tms5–2 mutants grown during a normal growing season. Figure S2. Pollen fertility and grain set of cv. YK17 and the T3 generation mutants (YK17S1 and YK17S2) plants grown under various temperature regimes. Figure S3. Phenotype of cv. YK17, YK17S1, R106 and the F1 hybrid of YK17S1 x R106. Figure S4. Phenotype of cv. YK17, YK17S1, R207 and the F1 hybrid of YK17S1 x R207. Figure S5. Grain yield performance and yield components of cv. YK17, YK17S1, R106 and the F1 hybrid YK17S1 x R106. Figure S6. Grain yield performance and yield components of cv. YK17, YK17S1, R207 and the F1 hybrid YK17S1 x R207. Figure S7. PCR-based identification of T-DNA-free in F1 progenies of YK17S1 x R101 using primers directed at the Cas9 (A), gRNA scaffold (B) and hpt (C) sequence. Figure S8. PCR-based identification of T-DNA-free in F1 progenies of YK17S1 x R106 using primers directed at the Cas9 (A), gRNA scaffold (B) and hpt (C) sequence. Figure S9. PCR-based identification of T-DNA-free in F1 progenies of YK17S1 x R207 using primers directed at the Cas9 (A), gRNA scaffold (B) and hpt (C) sequence. Table S1. Detection of mutations in potential off-target sites in T-DNA-free T1 generation segregants. Nucleotides corresponding to the protospacer adjacent motif in each target site are shown in red. Mismatches are shown in blue and matches in black. Table S2. List of primers used in the present study. (PDF 1285 kb) [file 12870_2019_1715_MOESM1_ESM.pdf]

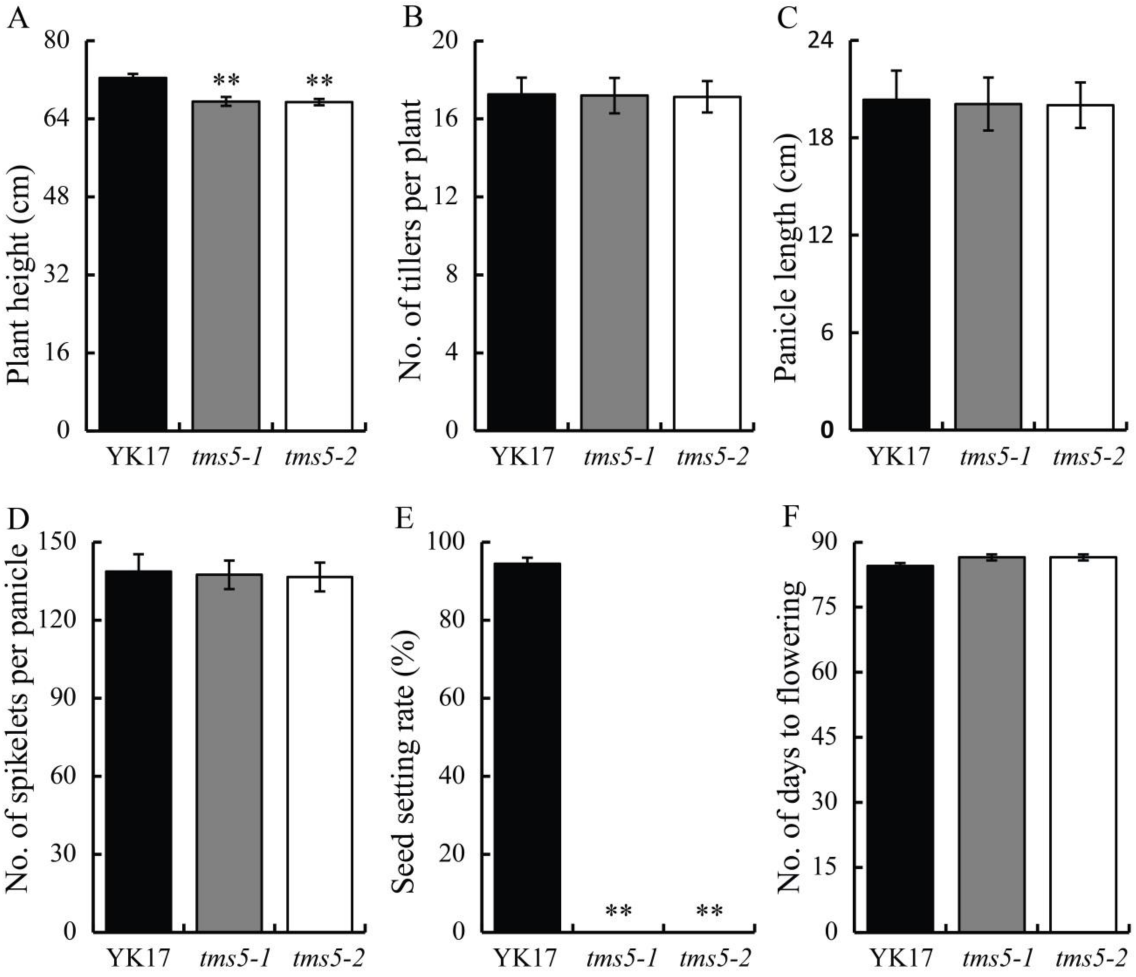

Figure S1. Agronomic performance of cv. YK17 and T_2 generation *tms5-1* and *tms5-2* mutants grown during a normal growing season. (A) Plant height. (B) Number of tillers per plant. (C) Panicle length (cm). (D) Number of spikelets per panicle. (E) Grain set. (F) Number of days to flowering. Values shown in the form mean ± SD (A-E: n=16, F: n=2). Asterisks (* P <0.05; ** P <0.01) indicate statistically significant differences between the mutant and cv. YK17 as determined by a student's t-test.

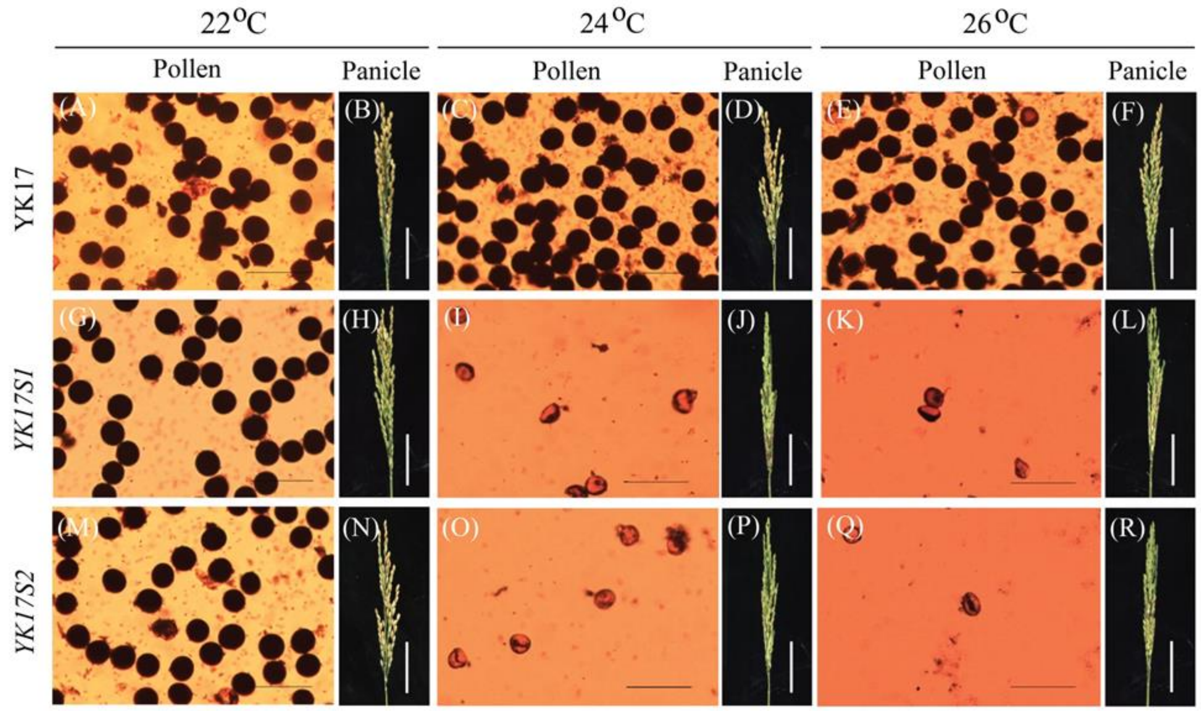

Figure S2. Pollen fertility and grain set of cv. YK17 and the T_3 generation mutants (YK17S1 and YK17S2) plants grown under various temperature regimes. (A-F) cv. YK17, (G-L) YK17S1, (M-R) YK17S2 exposed to 22 ℃, 24 ℃ or 26 ℃. Bars in (A, C, E, G, I, K, M, O, Q): 100 μm; in (B, D, F, H, J, L, N, P, R): 5 cm.

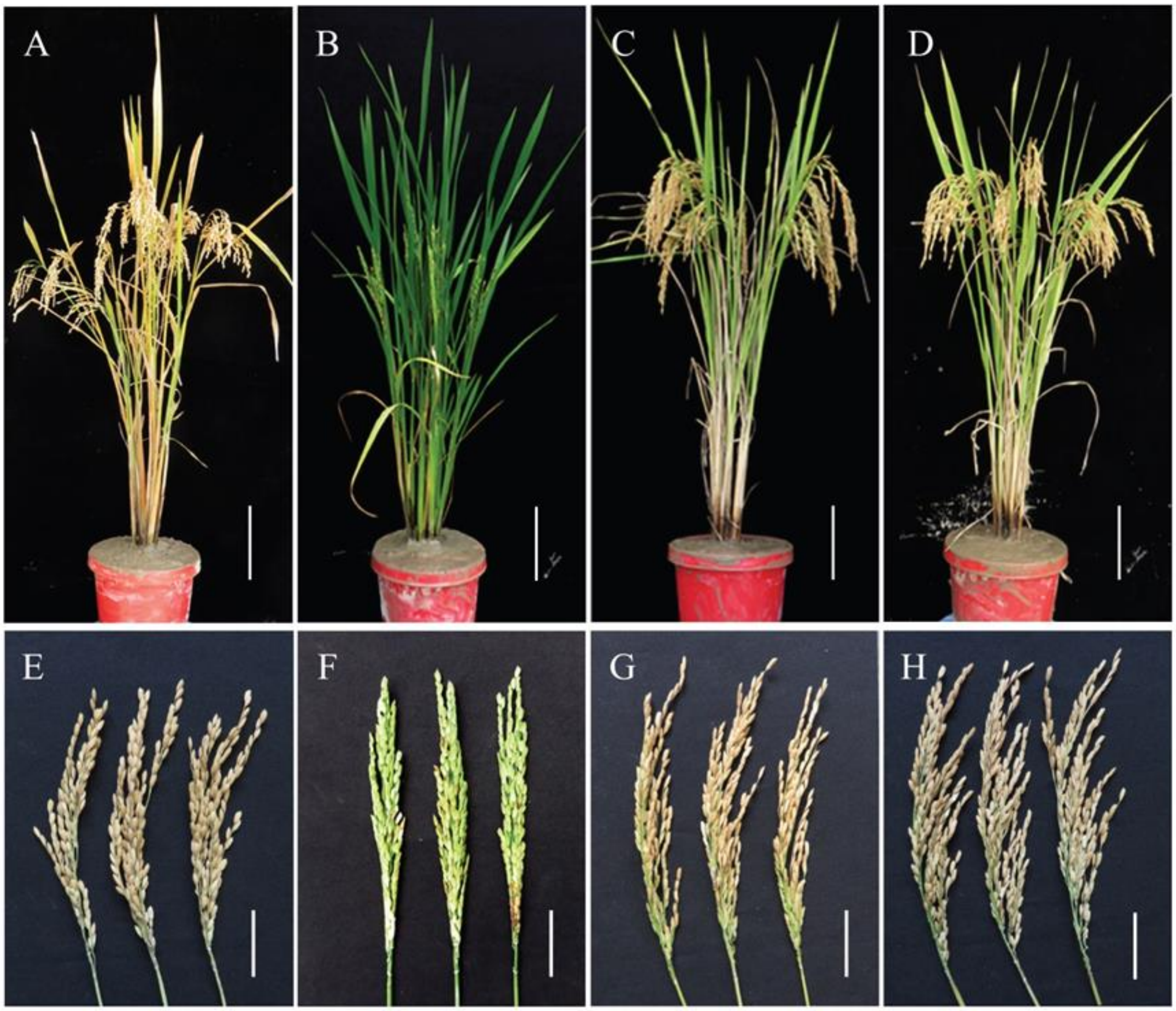

Figure S3. Phenotype of cv. YK17, YK17S1, R106 and the F_1 hybrid of YK17S1 x R106. Appearance of the (A-D) whole plant and (E-H) panicle of cv. YK17, YK17S1, R106 and the F_1 hybrid of YK17S1 x R106 from left to right, respectively. Bars in A-D: 15 cm, E-H: 5 cm.

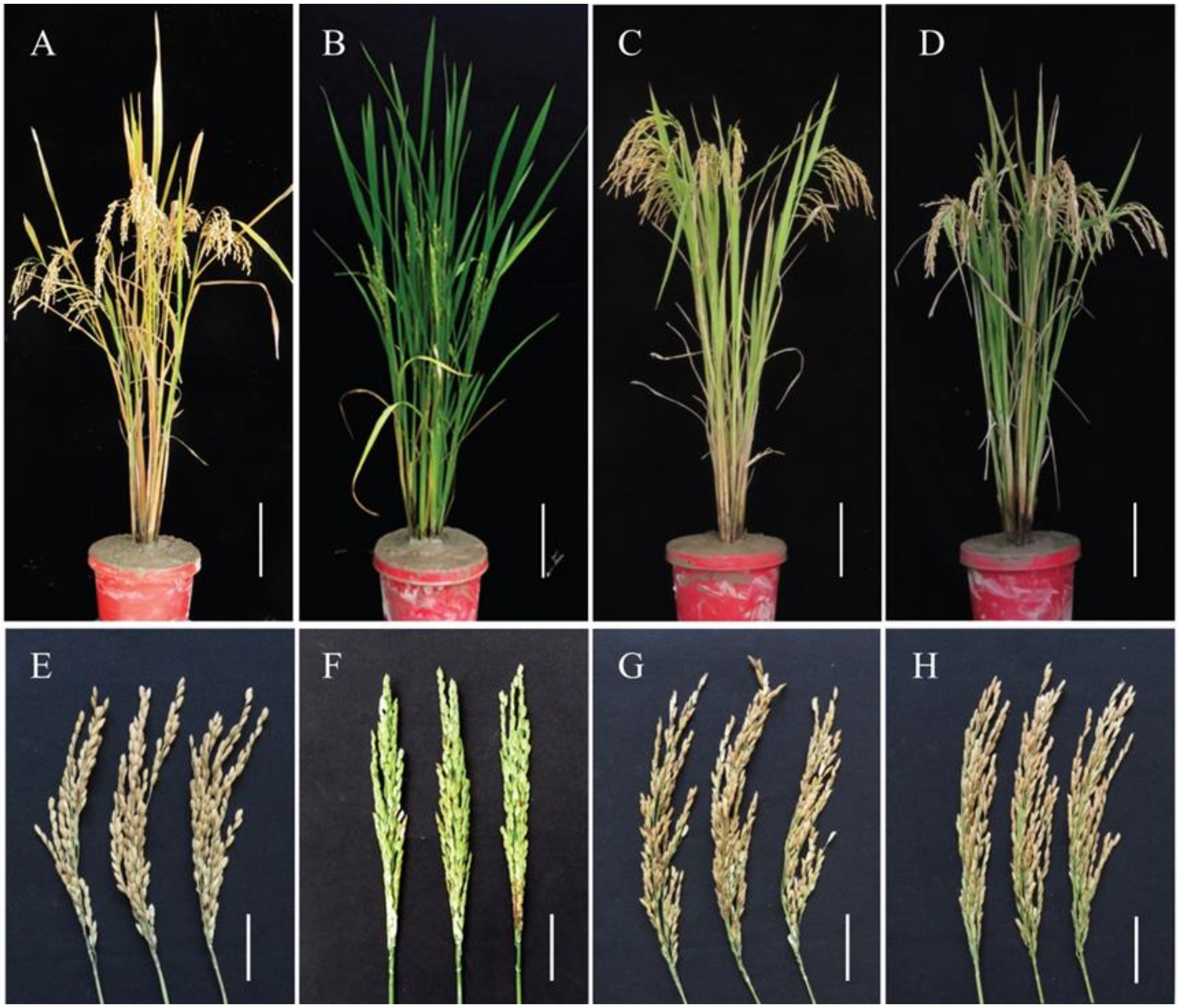

Figure S4. Phenotype of cv. YK17, YK17S1, R207 and the F_1 hybrid of YK17S1 x R207. Appearance of the (A-D) whole plant and (E-H) panicle of cv. YK17, YK17S1, R207 and the F_1 hybrid of YK17S1 x R207 from left to right, respectively. Bars in A-D: 15 cm, E-H: 5 cm.

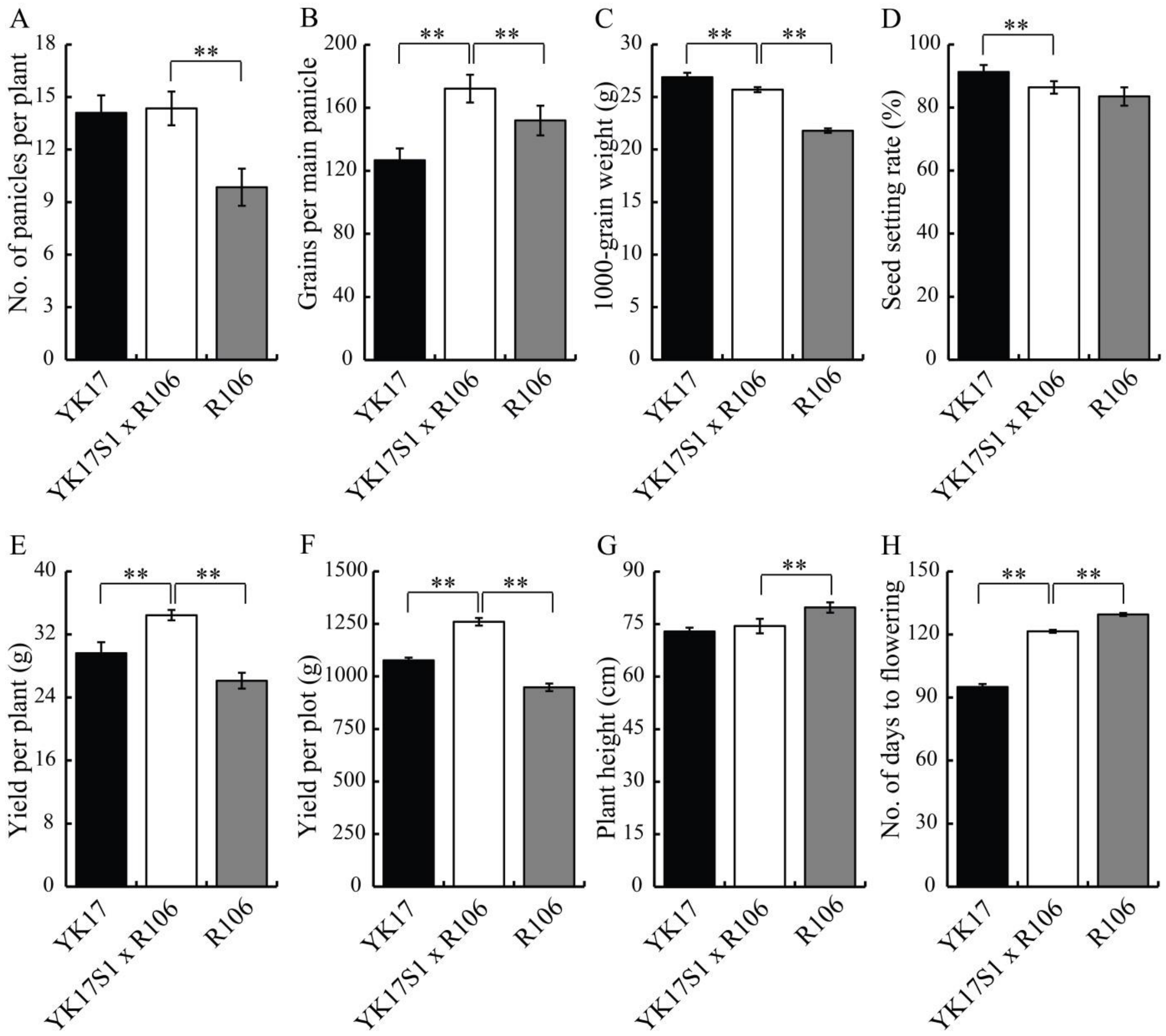

Figure S5. Grain yield performance and yield components of cv. YK17, YK17S1, R106 and the F$_1$ hybrid YK17S1 x R106. (A) Number of panicles per plant. (B) Number of grains set on the main panicle. (C) Thousand grain weight. (D) Grain set. (E) Yield per plant. (F) Yield per plot. (G) Plant height. (H) Number of days to flowering. Values shown in the form mean ± SD (A, B, D, G: *n*=20; C: *n*=4, E: *n*=6, F, H: *n*=2). Asterisks (* *P* <0.05; ** *P* <0.01) indicate statistically significant differences between the mean performances of either cv. YK17 and YK17S1 x R106, or R106 and YK17S1 x R106 as determined by a student's *t*-test.

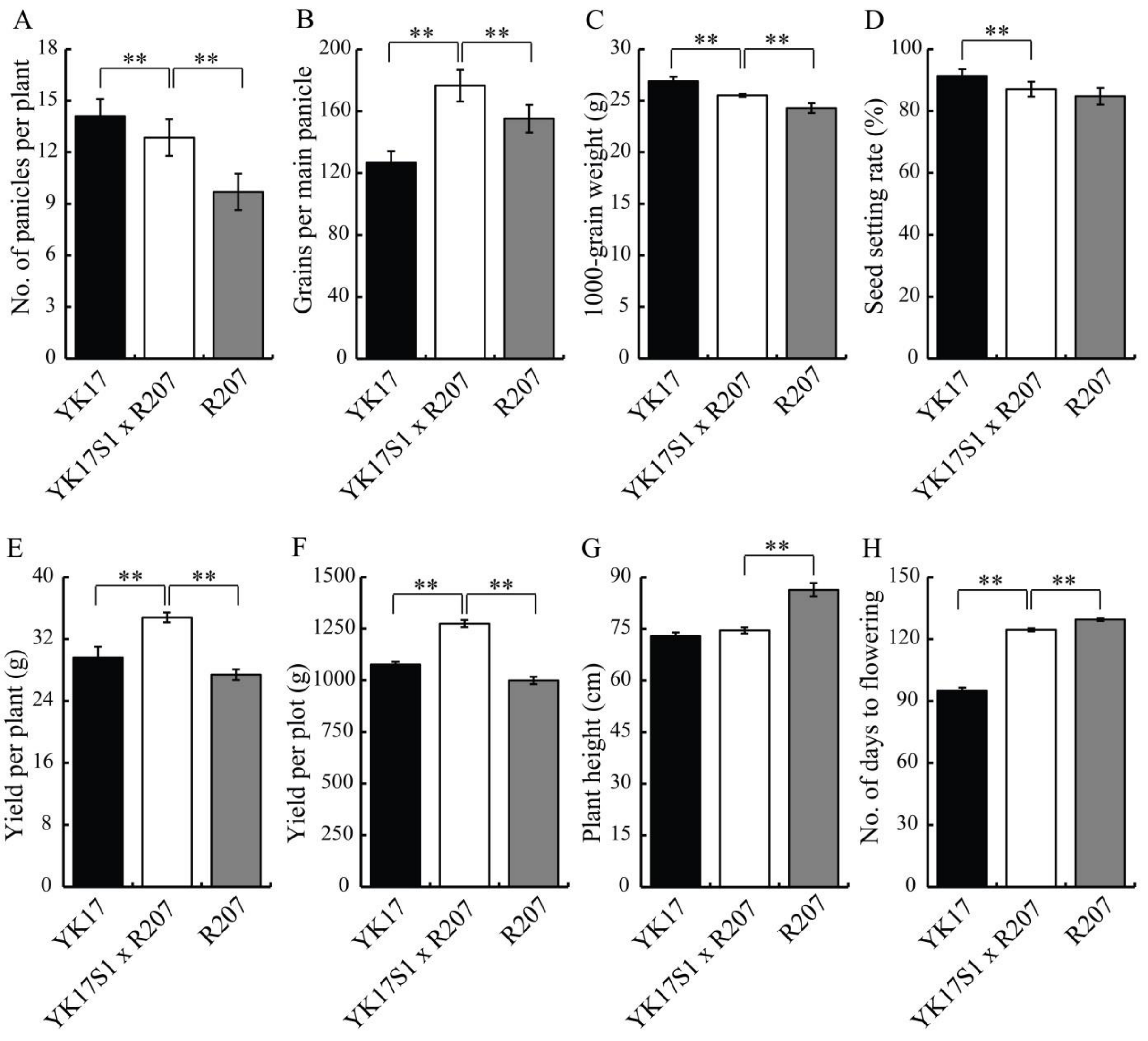

Figure S6. Grain yield performance and yield components of cv. YK17, YK17S1, R207 and the F_1 hybrid YK17S1 x R207. (A) Number of panicles per plant. (B) Number of grains set on the main panicle. (C) Thousand grain weight. (D) Grain set. (E) Yield per plant. (F) Yield per plot. (G) Plant height. (H) Number of days to flowering. Values shown in the form mean ± SD (A, B, D, G: *n*=20; C: *n*=4, E: *n*=6, F, H: *n*=2). Asterisks (* *P* <0.05; ** *P* <0.01) indicate statistically significant differences between the mean performances of either cv. YK17 and YK17S1 x R207, or R207 and YK17S1 x R207, as determined by a student's *t*-test.

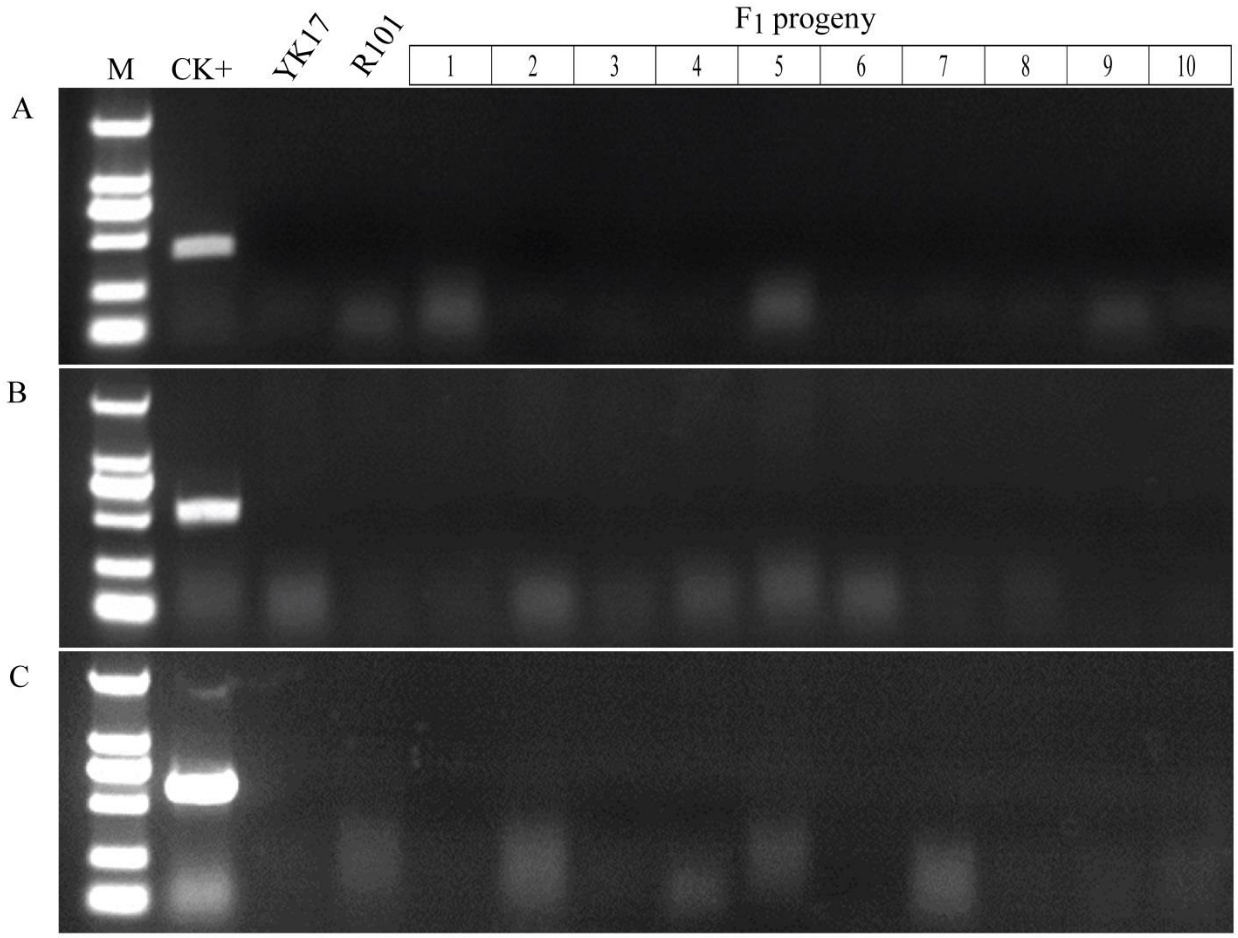

Figure S7. PCR-based identification of T-DNA free in F_1 progenies of YK17S1 x R101 using primers directed at the *Cas9* (A), gRNA scaffold (B) and *hpt* (C) sequence. The gDNA of transgene positive plant was used as positive control (CK+), and cv. YK17 and R101 as the negative control. M: 2 kbp DNA ladder.

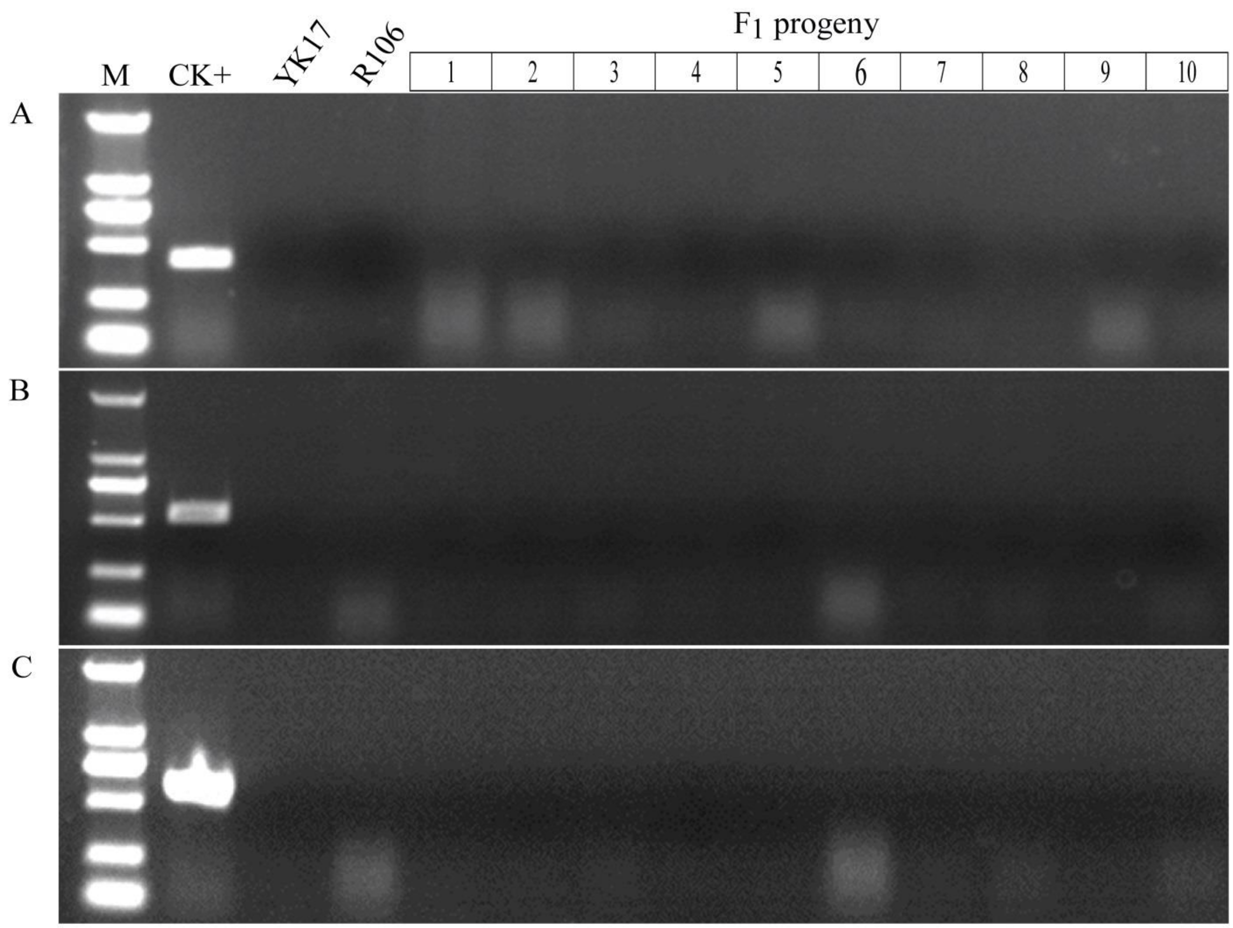

Figure S8. PCR-based identification of T-DNA free in F_1 progenies of YK17S1 x R106 using primers directed at the *Cas9* (A), gRNA scaffold (B) and *hpt* (C) sequence. The gDNA of transgene positive plant was used as positive control (CK+), and cv. YK17 and R106 as the negative control. M: 2 kbp DNA ladder.

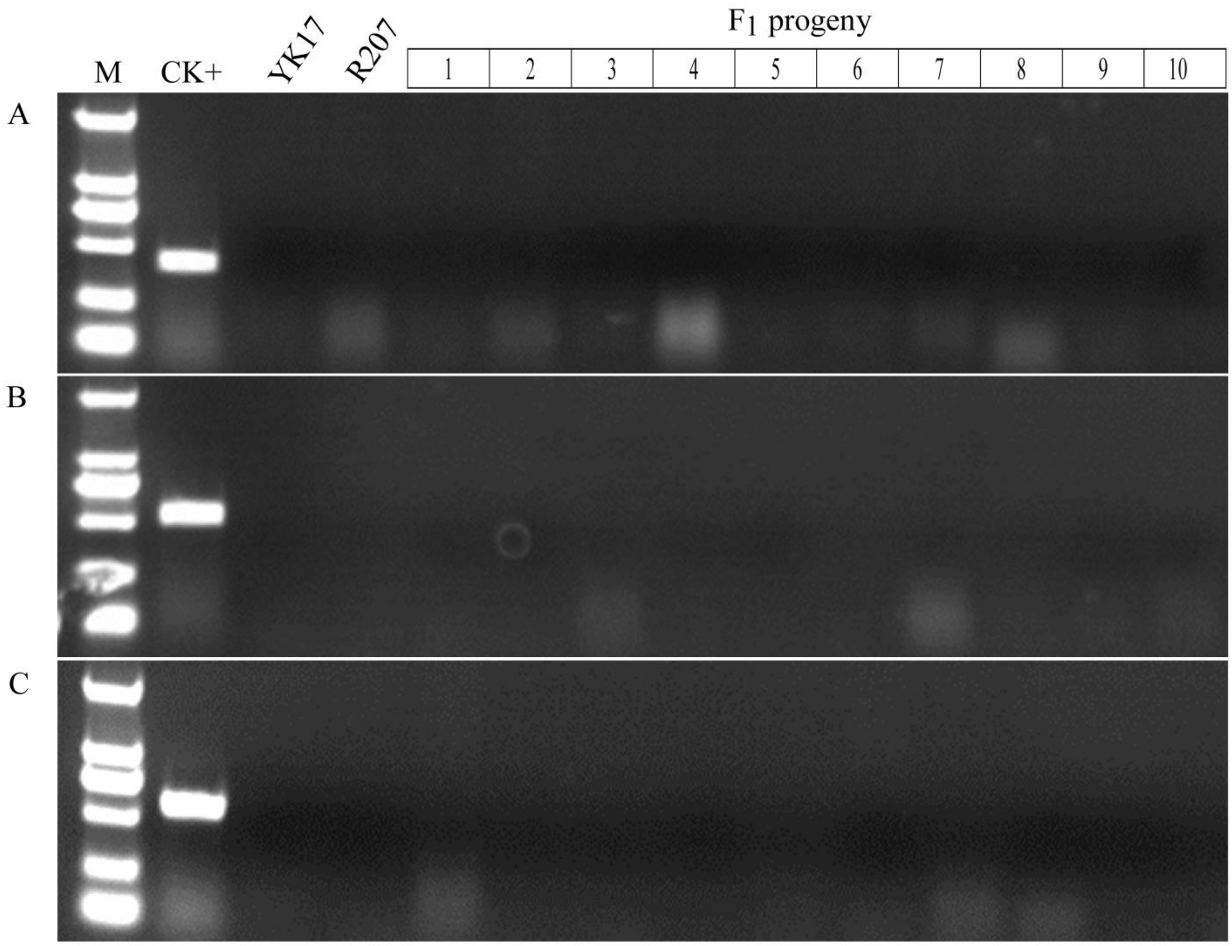

Figure S9. PCR-based identification of T-DNA free in F_1 progenies of YK17S1 x R207 using primers directed at the *Cas9* (A), gRNA scaffold (B) and *hpt* (C) sequence. The gDNA of transgene positive plant was used as positive control (CK+), and cv. YK17 and R207 as the negative control. M: 2 kbp DNA ladder.

Table S1. Detection of mutations in potential off-target sites in T-DNA-free T_1 generation segregants. Nucleotides corresponding to the protospacer adjacent motif motif in each target site are shown in red. Mismatches are shown in blue and matches in black.

Target site	Name of the putative off-target site	Position of the target site	Sequence of the target site	Number of mismatching bases	Number of plant sequenced	Number of plant with mutations
TMS5		Chr2: 6362762-6362784	CACCGTCGAGGGCTACCCCGTGG			
	TMS5-Chr1	Chr1: 2936471-2936493	TGGCTTCACGGGCTACCCCGTGG	6	5	0
	TMS5-Chr3	Chr3: 20468115-20468137	TGGCTTCATGGGCTACCCCGTGG	6	5	0
	TMS5-Chr8	Chr8: 8198377-8198399	TGTCTTCACGGGCTACCCCGTGG	6	5	0
	TMS5-Chr10	Chr10: 1035191-1035213	TGGCTTCACGGGCTACCCCGTGG	6	5	0

Table S2. List of primers used in the present study.

Primer name	Primer sequence (5'-3')	Purpose
TMS5V-F	CAGCACCGTCGAGGGCTACCCCG	Vector constructs
TMS5V-R	AACCGGGGTAGCCCTCGACGGTG	Vector constructs
Sqprimer	GATGAAGTGGACGGAAGGAAGGAG	Target sequencing
Cas9-F	TACTGAACTCCGAAATCTG	Genotyping
Cas9-R	CAACGGTGGCTTACTCT	Genotyping
gRNA-F	TGGTAGAAGTCGGAGATGT	Genotyping
gRNA-R	CTTTCCCTTTGTATTGCTG	Genotyping
HPT-F	TGCTCCATACAAGCCAACC	Genotyping
HPT-R	TGTCCTGCGGGTAAATAGC	Genotyping
TMS5S-F	CCATCGTGCTTCGTGCCAAAA	DNA sequencing
TMS5S-R	GCTCGTGCTCCTGACCAATCT	DNA sequencing
TMS5q-F	GCTCAGCCACAACCTCGTC	qRT-PCR
TMS5q-R	GCTGCTTGATCTCGCTCCC	qRT-PCR
Ubi-F	GCTCCGTGGCGGTATCAT	qRT-PCR
Ubi-R	CGGCAGTTGACAGCCCTAG	qRT-PCR
OsActin-F	CAATCGTGAGAAGATGACCC	cDNA-checking
OsActin-R	GTCCATCAGGAAGCTCGTAGC	cDNA-checking
Chr1-F	ATGGACGGAGTTGCTGC	DNA sequencing
Chr1-R	GTGGCGAATGCCTTTAGT	DNA sequencing
Chr3-F	GCGAAACTATCAACCAGC	DNA sequencing
Chr3-R	CGGATGTAATCTCAGAGGGA	DNA sequencing
Chr8-F	GATTGTTACGAGGACGATGT	DNA sequencing
Chr8-R	TGGATGCGGTGATAGCG	DNA sequencing
Chr10-F	CCATTCACCAGACCGCTAC	DNA sequencing
Chr10-R	CGGCTTTGGCATACTCATT	DNA sequencing
